# Multiparametric Detection of Effects of TILs and Oncolytic Virotherapy on Xenograft Mouse Model of Glioblastoma

**DOI:** 10.3390/biomedicines13122977

**Published:** 2025-12-04

**Authors:** Gaukhar M. Yusubalieva, Daria A. Chudakova, Polina G. Shirokikh, Diana V. Yuzhakova, Elena B. Kiseleva, Daria A. Sachkova, Varvara V. Dudenkova, Daria P. Kirsova, Maria S. Myzina, Elvira P. Yanysheva, Alexander V. Panov, Natalia F. Zakirova, Anastasia V. Poteryakhina, Alexander S. Semikhin, Alexander A. Kalinkin, Vladimir P. Baklaushev

**Affiliations:** 1 Federal Research and Clinical Center of Specialized Medical Care and Medical Technologies, Federal Medical and Biological Agency of Russia, 115682 Moscow, Russia; yuzhakova-diana@mail.ru (D.V.Y.); baklaushev@fccps.ru (V.P.B.); 2 Federal Center for Brain and Neurotechnologies, Federal Medical and Biological Agency of Russia, 117513 Moscow, Russiapolinasirokih@gmail.com (P.G.S.); dashakirsova@yandex.ru (D.P.K.); 3 Engelhardt Institute of Molecular Biology, Russian Academy of Sciences, 119991 Moscow, Russia; 4 Institute of Experimental Oncology and Biomedical Technologies, Privolzhsky Research Medical University, 603005 Nizhny Novgorod, Russia; kiseleva84@gmail.com (E.B.K.); sachkova.collins@gmail.com (D.A.S.);; 5 National Research Center for Epidemiology and Microbiology Named After Honorary Academician N.F. Gamaleya of the Ministry of Health of the Russian Federation, 123098 Moscow, Russia

**Keywords:** glioblastoma, animal models, immunotherapy, cell therapy, TILs, oncolytic viruses, optical coherence tomography (OCT), Fluorescence lifetime imaging microscopy (FLIM)

## Abstract

**Background/Objectives:** Glioblastoma (GBM) is an aggressive primary brain tumor with dismal prognosis and limited treatment options. Immunotherapy, including personalized approaches using tumor-infiltrating lymphocytes (TILs) and allogeneic natural (NK) or engineered killer cells (chimeric antigen receptor NK, NK-CAR), and oncolytic viruses (OV), has shown some potential in GBM. Combining different therapeutic strategies may enhance treatment efficacy. Here, we present a xenograft GBM mouse model with multiparametric detection for various immunotherapy research applications. **Methods:** In a xenograft GBM NOD-*Prkdcs* scid *Il2rg*em1/Smoc (NSG) mouse model based on orthotopic transplantation of patient-derived GBM cultures retaining tumor heterogeneity, intravenous and intratumor immunotherapeutic interventions by TIL and OV therapy were performed. Xenograft engraftment was evaluated using intravital MRI; delivery of OV and TILs to the tumor and changes in the tumor and peritumoral space were assessed using intravital confocal microscopy; and metabolic and structural changes in the tumor and peritumoral environment were assessed via fluorescence lifetime imaging microscopy (FLIM) and optical coherence tomography (OCT). The intravital imaging data were compared with the results of preliminary and final histological and immunocytochemical data. **Results:** Both OV and TILs demonstrated tumor-specific targeting and delivery across the blood–brain barrier. Further, we showed that in this model the xenograft response to both therapeutic treatments can be assessed using FLIM and OCT. **Conclusions:** Overall, this work presents an optimized mouse model suitable for assessing the effect of combined TIL immunotherapy and OV on GBM in translational studies.

## 1. Introduction

Glioblastoma multiforme (GBM) is the most common malignant primary brain tumor in adults. It is classified by the World Health Organization (WHO) as a grade IV tumor. GBM is characterized by rapid proliferation, aggressive invasiveness, therapeutic resistance, and high recurrence rate, resulting in a dismal prognosis with a median survival of approximately 12–15 months under the current standard of care. Currently, active research is focused on developing strategies to overcome therapeutic resistance in GBM.

Cancer immunotherapy (CI) is a curative approach aimed at activating the host immune system to target tumors or using cytokines and monoclonal antibodies as anti-cancer therapy. Although CI has demonstrated efficacy in various malignancies [1], its application in GBM might be challenging, mostly due to the marked cellular heterogeneity of the GBM tumor and its immunosuppressive tumor microenvironment (TME) [2]. Thus, combining multiple therapeutic strategies may enhance treatment efficacy. Overall, there are several promising approaches to GBM treatment under the umbrella of CI, including the use of immune checkpoint inhibitors, chimeric antigen receptor (CAR)-engineered T cells, CAR-engineered natural killer (NK) cells, as well as tumor-infiltrating lymphocytes (TILs)—autologous T cells isolated from a patient’s tumor, expanded ex vivo and infused back to the patient to boost the anti-tumor immune response. Further, the effectiveness of modified NK cells and TILs was previously investigated in primary human GBM cultures in vitro [3,4,5].

Additionally, oncolytic virotherapy has emerged as a curative approach to GBM. OV involves the use of genetically engineered (recombinant) replication-deficient viruses that selectively infect tumor cells and trigger their lysis while simultaneously stimulating an anti-tumor immune response and remodeling the immunosuppressive TME to be more “immune-vigilant” [6,7]. Several types of recombinant OV, such as Newcastle disease virus (rNDV), modified Herpes simplex virus (HSV), Adenovirus, Poliovirus, Parvovirus, and others have shown tumor-suppressive effects in GBM [8,9]. For example, one OV, DELYTACT (teserpaturev), genetically modified oncolytic HSV-1, is currently approved for GBM treatment; another one, DNX-2401 (tasadenoturev), genetically modified adenovirus, is undergoing clinical trials for the treatment of GBM, with rather encouraging results [10,11]. All of these findings support further development and clinical evaluation of OV therapy for GBM, including OV based on the use of recombinant poxviruses, including vaccinia viruses (VVs). In our previous work, we characterized several recombinant variants of the oncolytic VV, deficient in the viral thymidine kinase (*TK*) gene and encoding red fluorescent protein tagRFP and cytokines, as a potential anti-tumor OV in several syngeneic murine tumor models, such as melanoma (B16), colon cancer (MC38, CT26), and breast cancer (EO771) models [12]. Ablation of TK is a commonly used strategy to enhance the tumor-specificity of OV, as in non-cancerous cells TK is highly expressed only at the S-phase of the cell cycle, whereas in cancer cells its levels are constitutively high. Thus, the choice of this particular recombinant VV was justified by its enhanced specificity for tumor cells, resulting from the lack of TK, and by previously demonstrated efficiency in targeting highly immunosuppressive tumors (such as triple-negative breast cancer), allowing us to suggest its efficiency as a therapy for GBM.

Tumor modeling in mice has demonstrated a critical role in preclinical GBM research. However, despite recent advances in refining GBM models using various strains of immunodeficient mice, there are several limitations leading to restricted applicability of these models. Primary brain tumor modeling in animals has continuously improved over the past 60 years, with significant progress recently achieved through the development of highly invasive GBM animal models. Nevertheless, it is currently evident that no existing animal model fully recapitulates human GBM and its TME [13,14]. Our study aimed to identify a suitable GBM model for evaluating the effectiveness of immunotherapy combined with OV, and to comprehensively characterize such a model using multiparametric intravital imaging techniques. Here, we used NOD-*Prkdc* scid *Il2rg*em1/Smoc (NSG) mice, which do not have fully functional T, B, and NK cells. This permits efficient engraftment of xenograft tumors and provides a good platform for evaluating cell-based immunotherapy or OV on human GBM xenografts.

The array of multiparametric imaging techniques commonly used in in vivo studies includes magnetic resonance imaging (MRI), intravital confocal microscopy (IVM), lifetime fluorescence imaging (macro-FLIM and micro-FLIM), and optical coherence tomography (OCT). We previously applied some of these methods to study another xenograft GBM model based on the human glioblastoma cell line transduced with a bidirectional construct stably coexpressing luciferase (Luc2) and far-red fluorescent protein (mKate2) [15]. Notably, there are reports that luciferase expression can act as a tumor-associated antigen, eliciting an immune response against luciferase and influencing tumor growth [16], and internalization of the luciferase plasmid can alter the phenotype of recipient cells [17]. The same might be the case when labeling tumor cells with RFP proteins. The best approach for modeling GBM in vivo is using label-free xenograft cells.

FLIM (fluorescence lifetime imaging) is a label-free method used in optical diagnostics to assess changes in the intrinsic fluorescence of NAD(P)H, which indicate metabolic alterations, including in the context of GBM [18]. Considering the demonstrated potential of OCT in neurosurgery for real-time evaluation of tissue microstructure [19], it is important to adapt both of the aforementioned methods as rapid, non-invasive tests for assessing the therapeutic efficacy of GBM treatment.

The overall goal of our study was to develop a patient-derived xenograft GBM NSG mice model enabling non-invasive multiparametric assessment of the effects of TILs and OV therapy for individual patients. To achieve this goal, we aimed to optimize approaches to patient-derived xenograft in vitro cultures, implantation of human xenograft GBM to NSG mice, follow-up non-invasive monitoring, and structural and metabolic characterization of tumor and peritumoral tissue, including for evaluation of therapy using OV and TILs. Overall, our work proves the xenograft GBM NSG mouse model to be suitable for studying VV-based OV therapy by LIVP-RFP as well as cell-based immunotherapy by TILs, and for assessing the metabolic and structural changes evoked by the aforementioned therapies in a given model.

## 2. Materials and Methods

### 2.1. Patients

The collection and use of patient tumor tissues were conducted in the Neurosurgical department of the Federal Scientific Center of the Federal Medical and Biological Agency and the Federal Center of Medical Sciences of the Federal Medical and Biological Agency from patients with diagnosis of glioblastoma in 2017–2024, in accordance with the Local Ethics Committee protocol. Prior to participation, all patients provided written informed consent in accordance with the Declaration of Helsinki. The diagnosis of GBM was confirmed by subsequent histopathological analysis conducted by pathologists.

### 2.2. Patient-Derived GBM Cells

Surgically removed tumor specimens from patients with GBM undergoing tumor resection surgery were immediately placed on ice in culture media and processed as soon as possible. In sterile conditions, samples were rinsed with Dulbecco’s Phosphate-Buffered Saline (DPBS) and minced with a scalpel to obtain 1–2 mm fragments. To prevent drying of the tissue, DPBS was added to cover the specimen. The mixture of tissue fragments was passed through a 100 µm pore sieve into a 50 mL centrifuge tube, separating the cell suspension from large tissue fragments. The suspension was distributed into two tubes of 15 mL each and centrifuged for 5 min at 500 rpm. The supernatant was removed, and the cell pellet was collected.

Samples were then divided into two parts, to be cultured either in Dulbecco’s Modified Eagle Medium-F12 (DMEM) supplemented with 10% Fetal Bovine Serum (FBS) or in Human Plasma-Like Medium (HPML) (Thermo Fisher, Waltham, MA, USA). Cells were cultured in a 6-well plate at 37 °C in a humidified incubator with 5% CO_2_ and either ~18.6% O_2_ (normoxia) or 5% O_2_ (hypoxia) and routinely tested for mycoplasma using Myco Real-Time kit (Evrogen, Moscow, Russia); media was replaced every 2–3 days. Expanded cells were cryopreserved following standard protocols and stored in liquid nitrogen until further use.

### 2.3. TILs

Isolation and purification of TILs, their phenotypic characterization, and the ex vivo expansion and activation of CD4+ and CD8+ cytotoxic anti-tumor lymphocytes were carried out according to a previously developed and described protocol [5]. To investigate the timing of TIL delivery to the tumor via IVM, both intratumoral and intravenous administrations of TILs were performed. For the study of the metabolic status in the tumor and peritumoral environment by the FLIM imaging and OCT, intra-tumoral administration of TILs was performed to exclude systemic effects (Table 1).

### 2.4. Animals

The study was carried out in compliance with the ARRIVE guidelines. NOD-*Prkdc* scid *Il2rg*em1/Smoc (NSG) line mice, males, 6–8 weeks old, were used in this study. The animals were obtained from the Laboratory Animal Breeding Facility of the Institute of Bioorganic Chemistry, Russian Academy of Sciences. All procedures with mice were performed under barrier conditions in strict adherence to institutional and national ethical guidelines for the care and use of laboratory animals. Initially, all animals were randomly divided into two main groups: implantation in the cortex and in the striatum. In each group (cortex or striatum), the animals were randomly assigned to subgroups based on therapy: OV (LIVP-RFP) or TILs (Table 2).

### 2.5. Xenograft Model of GBM

To model GBM in vivo in a mouse model, patient-derived primary cells were thawed, washed from cryopreservation buffer, and grown to a monolayer as described above. Before implantation to animals, the cell suspension was prepared by gently detaching cells using TrypLE Express solution (Thermo Fisher Scientific, Waltham, MA, USA), as per standard protocol. An automated cell counter ADAM™ CellT (NanoEntec, Seoul, Republic of Korea) and staining with propidium iodide were used to count the cells and assess their viability using propidium iodide. The animals were pre-anesthetized using a solution of Zoletil (Virbac Sante Animale, France) 40 mg/kg, 50 µL mixed with Rometar (Bioveta, Check Republic), 10 mg/kg, diluted in sterile water. The animals were pre-anesthetized using a solution of Zoletil (Virbac Sante Animale, Carros, France), 40 mg/kg, mixed with 50 µL Rometar (Bioveta, Ivanovice na Hané, Czech Republic), 10 mg/kg, diluted in sterile water. To prepare the surgical field after fixing the animal, scalp shaving and antiseptic treatment of the scalp were performed. Next, using a stereotaxic system and microsurgical instruments, a trephination hole was made in the skull over the projection of the striatum of the right brain hemisphere. The coordinates for the trephination were pre-calculated using a stereotaxic mouse brain atlas [20] to ensure precise injection of the cell suspension into the target area. The GBM cell suspension, containing 3 × 10^5^ cells in 5 µL of sterile DPBS, was injected into the right hemisphere of the brain of mice (*n* = 9), according to the stereotaxic coordinates AP + 1.0 mm, ML + 1.5 mm, and DV − 2.5 mm, and into the cortex of the brain of mice (*n* = 9) according to the following coordinates: AP − 2.0 mm, ML + 1.5 mm, and DV − 1.2 mm. An RWD injector at a rate of 1 µL/min was used, minimizing the risk of brain tissue damage and ensuring even cell distribution. After the cell suspension was injected, the needle was kept in the brain tissue for 5 min to prevent backflow. Then, the surgical site was aseptically closed by applying a mattress suture to the scalp. The suture was treated with an antiseptic to prevent infection and promote healing. For several days after the surgery, the animals were closely monitored to assess their condition, including evaluation of activity and postoperative wound healing.

### 2.6. Recombinant Variant of the Oncolytic Vaccinia Virus Lister Strain

Recombinant variants of oncolytic vaccinia virus (VV) of the Lister strain, developed at the Moscow Institute of Viral Preparations (LIVP), featuring deletions in the thymidine kinase (TK) gene to enhance tumor cell specificity, and encoding various chemokines and cytokines to augment the immune response, were created by the research team led by Prof. P.M. Chumakov at the Engelhardt V.A. Institute of Molecular Biology of the Russian Academy of Sciences [21], and were kindly provided to us. For tracking targeted delivery to tumors, a LIVP strain with TK deletion and expressing the reporter gene tagRFP under the control of the 7.5 K promoter (LIVP-RFP) was constructed, successfully tested as a control virus in vitro in a previously published study [22]. Expression of tagRFP in infected cells was confirmed via fluorescence microscopy. All recombinant viruses were purified by gradient sucrose centrifugation for in vitro and in vivo experiments. Viral titers were determined as previously described [22].

For the study of the metabolic status in the tumor and peritumoral environment using FLIM imaging and OCT, intratumoral administration of the virus was performed to exclude systemic effects, at the dose according to Table 1.

### 2.7. Immunocytochemical Analysis (ICC)

For ICC, the primary GBM cells were cultured on chamber slides (Biologix, Shandong, China). After forming a cell monolayer, the medium was removed and the cells were washed twice with DPBS and fixed with 4% paraformaldehyde for 15 min at room temperature. Following fixation, the cells were washed twice with DPBS and incubated for 30 min at room temperature in a blocking buffer (DPBS with 5% FBS and 0.3% Tween-20) on a shaker to prevent nonspecific antibody binding. Next, the cells were washed with DPBS with 0.3% Tween-20 (washing buffer) and incubated overnight at 4 °C with primary antibodies Nestin (A11861, ABclonal, Wuhan, China), Sox-2 (A0561, ABclonal, Wuhan, China), and pab-GFAP (Z0334, Dako, Glostrup, Denmark) diluted 1:100 in blocking buffer. Cells were then washed three times (10 min each) with washing buffer, incubated for 1.5 h with fluorophore-conjugated secondary antibodies (anti-goat A-11034 and anti-rabbit A-11034, from Invitrogen, Waltham, MA, USA), and washed with DPBS. Nuclear staining was performed with Hoechst dye (1:100) for 3 min, followed by three DPBS washes. Imaging was performed via confocal microscopy (Eclipse Ti2 A1R, Nikon, Tokyo, Japan).

### 2.8. Quantitative Reverse Transcription Polymerase Chain Reaction (qRT-PCR)

Total RNA was isolated using the ExtractRNA kit (Evrogen, Moscow, Russia) per the manufacturer’s instructions. Residual genomic DNA was removed using the DNase E kit (Evrogen). The concentration of RNA was determined by the NanoDrop ND-1000 spectrophotometer (Thermo Fisher Scientific, Waltham, MA, USA). Equal RNA amounts of 1 μg were reverse-transcribed to cDNA using the Reverta L kit (AmpliSens, Moscow, Russia) following the manufacturer’s protocol. qRT-PCR was performed on a Roche LightCycler 96 (Roche, Basel, Switzerland) using 2 μL cDNA and reaction mix qPCRmix-HS SYBER (Evrogen).

Primer sequences used are as follows:

Oct4 forward 5′-AGCAAAACCCGGAGGAGT-3′, reverse 5′-CCACATCGGCCTGTGTATATC-3′; COX2 forward 5′-GCCAAGCACTTTTGGTGGAG-3′, reverse 5′-GGGACAGCCCTTCACGTTA-3′; Notch1 forward 5′-CCTGAGGGCTTCAAAGTGTC-3′, reverse 5′-CGGAACTTCTTGGTCTCCAG-3′; CD90 forward 5′-GTTAGGCTGGTCACCTTCTG-3′, reverse 5′-GAGATCCCAGAACCATGAACC-3′; CD44 forward 5′-ATGGACAAGTTTTGGTGGCA-3′, reverse 5′-TTACACCCCAATCTTCATGTC-3′; vimentin forward 5′-AGATGGCCCTTGACATTGAG-3′, reverse 5′-CCAGAGGGAGTGAATCCAGA-3′; COX2 forward 5′-GCCAAGCACTTTTGGTGGAG-3′, reverse 5′-GGGACAGCCCTTCACGTTA-3′; N-cadherin forward 5′-TGGAACGCAGTGTACAGAATCAG-3′, reverse 5′-TTGACTGAGGCGGGTGCTGAATT-3′, Nox1 forward 5′-CAATCTCTCTCCTGGAATGGCATCCT-3′, reverse 5′-CCTGCTGCTCGGATATGAATGGAGAA-3′; Nox4 forward 5′-CTGCATGGTGGTGGTGCTAT-3′, reverse 5′-CCGGGAGGGTGGGTATCTAA-3′; Notch2 forward 5′-CCAGACATTCTTGCAGCTTGGACT-3′, reverse 5′-GGCATAATTCCCAACAGGACGCTA-3′, Nanog forward 5′-ATGCCTCACACGGAGACTGT-3′, reverse 5′-AGGGCTGTCCTGAATAAGCA-3′; Nox5 forward 5′-ATCTGCTCCAGTTCCTGCAT-3′, reverse 5′-ACAAGATTCCAGGCACCAG-3′; COX7A2 forward 5′-CTCGGAGGTAGTTCCGGTTC-3′, reverse 5′-TCTGCCCAATCTGACGAAGAG-3′; GUSB (housekeeping gene) forward 5′-CGTGGTTGGAGAGCTCATTTGGAA-3′, reverse 5′-ATTCCCCAGCACTCTCGTCGGT-3′.

The amplification program was as follows: amplification at +95 °C for 5 min, followed by 40 cycles of amplification: denaturing at +95 °C/10 s; annealing at +55 °C/20 s; elongation at +72 °C/20 s, followed by melting curve analysis step +95 °C/5 s, +65 °C/1 s, and +97 °C/1 s. Reaction specificity was validated by melting curve analysis and agarose electrophoresis of PCR products. No-template controls were included. Data were analyzed using the standard 2^−ΔΔCt^ method with normalization to reference housekeeping gene; data were presented as fold change relative to DMEM normoxia. All qPCR reactions were performed in at least duplicate.

### 2.9. In Vivo Magnetic Resonance Imaging (MRI) and Analysis

To assess the engraftment of GBM cells in mice, regular monitoring was performed using a high-field MRI scanner designed for small laboratory animals (ClinScan 7T; Bruker Biospin, Ettlingen, Germany) at the Core Facility “Medical and Biotechnological Nanotechnologies” of the Russian National Research Medical University named after N. I. Pirogov, Moscow. Scanning was performed under inhalation anesthesia (1.5% isoflurane in an oxygen mixture at a flow rate of 250 mL/min) applied using the EZ-7000 Classic System (E-Z Systems Inc., Boring, OR, USA). T2-weighted images in axial, coronal, and sagittal planes were obtained using a coil designed for the brains of small laboratory animals.

### 2.10. Intravital Confocal Microscopy (In Vivo Microscopy, IVM)

IVM was performed on a Nikon A1 MP confocal microscope (Nikon, Tokyo, Japan) equipped with a heated adapter stage optimized for use with 50 mm diameter round cover glasses. Mice with superficially located tumors in the cerebral cortex, confirmed by MRI, were anesthetized with Rometar (50 µL, 8 mg/mL) and Zoletil (19 mg/kg).

In sterile conditions, an intracranial window was created by thinning and removing a portion of the parietal bone over the region where the glioblastoma was localized, and the animal was placed on the stage so that the area of interest was in close contact with the cover glass surface and accessible for microscopy. To assess the overall infiltration of the tumor microenvironment by immune cells and the expression of PD1, 15 min prior to IVM, 2 μL of labeled antibodies (Brilliant Violet 421 anti-mouse CD45 antibody; # 103134, BioLegend, San Diego, CA, USA or APC anti-PD1 antibody; # 109112, Biolegend, San Diego, CA, USA) diluted in 100 μL DPBS was injected into the tail vein. The recruitment of myeloid cells was assessed by intravenous administration of primary labeled anti-CD11b (101208, BioLegend, San Diego, CA, USA) and anti-CD11c (117308, BioLegend, San Diego, CA, USA), and tumor neoangiogenesis was studied using anti-CD105 (120420, BioLegend, San Diego, CA, USA). To track the release of immune cells from the vascular bed, mice were injected with 2 μL of a solution of labeled anti-CD31-Alexa700 (# 303134; BioLegend, San Diego, CA, USA). For the assessment of delivery to the tumor, LIVP RFP and TILs were administered intravenously via tail vein injection in 100 µL of physiological saline at the dose specified in Table 2. To track the signal from TILs in vivo, the latter was labeled with the vital tracer eBioscience™ Cell Proliferation Dye eFluor™ 670 (excitation peak 633 nm, emission peak 670 nm) allowing for tracing cells through several cell divisions (Invitrogen, Waltham, MA, USA). Labeled cells were administered as a single slow infusion dose using an infusion pump at a rate of 10 μL/min. The delivery of the oncolytic virus LIVP-RFP to the tumors was tracked by detection of RFP fluorescence. The localization of labeled cells relative to the tumor focus was determined by stepwise Z-axis scanning. Several tumor regions were scanned sequentially over 120 min (1 frame at 512 × 512 or 1024 × 1024 pixels every 40–60 s). All acquired images were then combined into a stack, generating a 3D tissue model. Upon completion of IVM, the intracranial window was covered with a skin flap, a mattress suture was applied, and the area was treated with an antiseptic solution. Confocal microscopy images were processed using NIS Elements AR software version number 5.21 (Nikon, Tokyo, Japan). The localization of labeled cells relative to the tumor site was determined using stepwise imaging with incremental movements along the Z-axis. Multiple tumor sections were scanned sequentially over 120 min (1 frame of 512 × 512 or 1024 × 1024 pixels every 40–60 s). All acquired images were then combined into a stack, creating a 3D tissue model.

### 2.11. Histology and Immunohistochemical (IHC) Analysis

Animals were deeply anesthetized using a solution of Zoletil (40 mg/kg, Virbac Sante Animale, France) in combination with Rometar (10 мг/кг; Bioveta, Ivanovice na Hané, Czech Republic). This ensured a humane and painless procedure. Next, transcardial perfusion was performed using a 4% paraformaldehyde solution for tissue fixation. After perfusion, the animals were decapitated and the brain was extracted using microsurgical instruments and placed in 4% paraformaldehyde for fixation for 48 h at +4 °C.

For IHC analysis of the brain tissue, 50 µm thick brain sections were prepared using a vibratome Microm HM 650V 5100mz (Campden Instruments, Loughborough, UK) after fixation. The obtained sections were incubated for 30 min in Dulbecco’s Phosphate-Buffered Saline (DPBS) containing 0.1% Tween-20, 0.05% Triton X-100, and 2% mouse serum, and next with primary antibodies against Sox2 (A0561, ABclonal, Wuhan, China), Nestin (A11861, ABclonal, Wuhan, China), GFAP (Z0334, Dako, Glostrup, Denmark), PECAM-1 CD31 (A0378, ABclonal, Wuhan, China), and PD-1 (109112, Bilegend, San Diego, CA, USA) diluted 1:100 in DPBS overnight at +4 °C. For IHC, antibodies conjugated to fluorophores against mouse CD45 (103134, BioLegend, San Diego, CA, USA), CD49b (108912, BioLegend, San Diego, CA, USA), CD105 (120420, BioLegend, San Diego, CA, USA), CD140b (136008, BioLegend, San Diego, CA, USA), CD11c (117308, BioLegend, San Diego, CA, USA), CD11b (101208, BioLegend, San Diego, CA, USA), CD8 (100753, BioLegend, San Diego, CA, USA), CD206 (141717, BioLegend, San Diego, CA, USA), NK1.1 (108731, BioLegend, San Diego, CA, USA), and CD19 (115549, BioLegend, San Diego, CA, USA) were also used at a dilution of 1:100. Following primary antibody incubation, sections were washed three times with DPBS containing 0.1% Tween-20 and 0.05% Triton X-100, with each wash ~10 min. Next, sections were incubated for 1.5–2 h at room temperature in the dark on shaker with secondary antibodies conjugated with goat Alexa Fluor 488 (2541675, Thermo Fisher Scientific, Waltham, MA, USA) or donkey Alexa Fluor 555 (A31572, Thermo Fisher Scientific, Waltham, MA, USA), diluted 1:200 in 100 uL DPBS. For the staining of cell nuclei, brain sections were incubated in DPBS solution with Hoechst at a dilution of 1:1000 (1 μg/mL) in the dark for 10–15 min. Next, sections were washed three times in a large volume of DPBS.

Staining of paraffin sections for Ki67 (790-4286, clone 30-9, rabbit monoclonal, Roche, Basel, Switzerland), S-100 (760-2523, rabbit polyclonal, Roche, Basel, Switzerland), GFAP (EP672Y, rabbit monoclonal, Roche, Basel, Switzerland), and CD8 (790-4460, rabbit monoclonal, Roche, Basel, Switzerland) was performed in a Benchmark Ultra Immunostainer (Roche, Basel, Switzerland) using the primary antibodies and the OptiView DAB IHC detection kit (Roche, Basel, Switzerland) according to the manufacturer’s protocols. Stained and coverslipped slides were scanned with a Leica Aperio GT450 DX scanner (Leica Biosystems, Nussloch, Germany) and processed at 20× magnification using Aperio ImageScope software version number 12.3 (Leica Biosystems, Nussloch, Germany).

### 2.12. Fluorescence Microscopy

Fluorescence from both exogenous and endogenous fluorophores was detected using an LSM 880 confocal laser scanning microscope (Carl Zeiss, Oberkochen, Germany). Excitation of different molecules was performed with a Ti:Sa femtosecond laser MaiTai HP (Spectra-Physics Inc., Milpitas, CA, USA) with an 80 MHz repetition rate of 120 fs duration pulses. The images were obtained using a water immersion lens, C-Apochromat 40×/1.2 NA. Autofluorescence excitation of reduced nicotinamide adenine dinucleotide nucleotide (phosphate) (NAD(P)H) was conducted in a two-photon regiment at a wavelength of 750 nm with detection in the range of 450–490 nm (470/40 bandpass filter).

Fluorescence of red fluorescent protein RFP from LIVP-RFP was obtained for brain samples with GBM to identify the tumor zone on FLIM images. The RFP signal was excited by a picosecond diode laser (BDS-594-SM, Becker & Hickl GmbH, Berlin, Germany) at a wavelength of 594 nm with power incident on the sample of 5 µW, and detected at a wavelength of 610 nm, determined by a long-pass filter (610 LP, Chroma, Vermont, BF, USA). Fluorescent images of the far-red fluorescent protein Dye670 from labeled TILs were obtained for brain samples with GBM to identify the tumor zone on FLIM images. The Dye670 signal was excited at a wavelength of 633 nm by a picosecond diode laser, and detected at a wavelength of 670 nm.

### 2.13. Fluorescence Lifetime Imaging Microscopy (FLIM)

A confocal laser scanning microscope LSM 880 equipped with a time-correlated single photon counting (TCSPC) module (Becker & Hickl GmbH, Berlin, Germany) was used to obtain time-resolved microscopic fluorescence images of NSG brain tissue samples. Autofluorescence was excited at the wavelength of 750 nm with a femtosecond laser MaiTai HP (Spectra-Physics Inc., Milpitas, CA, USA) and detected in the range of 450–490 nm (Chroma, USA). Image acquisition time was 60 s. The average laser power on the samples was 6 mW. For each sample, 4–6 FLIM images were acquired.

The fluorescence decays of NAD(P)H were processed in the SPCImage software version number 8.9 (Becker & Hickl GmbH, Berlin, Germany). On average, 5000–10,000 photons were collected per decay curve (binning factor 2–4) and the fitting was performed using a bi-exponential decay model. The average value of the approximation quality X2 was from 0.8 to 1.2. The values of the short and long components of the lifetimes (t1 and t2) and their relative contributions (a1 and a2, a1 + a2 = 100%) were obtained, which correspond to the free and protein-bound forms of the NAD(P)H cofactor, respectively. The amplitude-weighted average lifetime was calculated as tm = (a1 × t1 + a2 × t2)/(a1 + a2). In the tumor images, fluorescence lifetimes were analyzed in cell cytoplasm by manual selection of the maximal area of cytoplasm as the region of interest in each individual cell. For each mouse, FLIM tumor images were acquired from 4–6 fields of view, for a total of 50–60 cells. For images displaying peritumoral and normal white matter, decay parameters were estimated in 10–12 regions of microscopic fields of view.

### 2.14. Statistical Analysis

Statistical analysis was conducted in GraphPad Prism version number 8.0.2 (GraphPad, San Diego, CA, USA) using built-in functions. Data were presented as mean values (M) ± standard deviation (SD). Statistical analyses were performed using unpaired *t*-tests and 2-way ANOVA. Differences were considered significant when *p* < 0.05. Data analysis and visualization of qRT-PCR data were performed using R (tidyverse, ggplot2) and presented as mean values (M) ± SEM.

### 2.15. OCT

Following the FLIM analysis, the next step was to study the optical properties of the tumor during its natural growth and after therapy by assessing backscattered radiation from the samples using OCT.

A spectral-domain multimodal OCT device (Institute of Applied Physics of Russian Academy of Sciences, Nizhny Novgorod, Russia) was used. The device operates on 1310 nm and has a common-path interferometric layout. The axial resolution is 10 μm and the transverse resolution is 15 μm in air. The scanning rate of the device is 20,000 A-scans/s, which allows 3D visualization of 2.4 × 2.4 × 1.25 mm^3^ tissue volume in 26 s with the simultaneous building of 2D OCT images in three different planes: along the scanning plane, perpendicular to the scanning plane, and en face images (top view—256 × 256 A-scans).

Three-dimensional OCT images were acquired in contactless mode. The entire surface of the sample was scanned line by line with a step of 2 mm to ensure overlap between adjacent OCT images for ease of subsequent image stitching. The obtained 3D OCT images were quantitatively analyzed—converted into 2D en face color-coded images of the attenuation coefficient distribution as described in [23]. The attenuation coefficient (μ) was calculated in each A-scan in the depth range 120–300 μm to obtain the highest contrast color-coded maps and provide the best information about the morphology of brain tissue, which was shown in our previous study [24].

In en face maps, the orange and red colors represent the areas with high μ values; blue represents low μ values; and azure, green, and yellow represent intermediate ones, respectively. By comparing the resulting color-coded maps of the scanned brain sample and histological sections, we were able to accurately determine the tumor location. Next, three regions of interest (ROIs) on each color-coded brain map were selected for calculation of the mean values of μ, tumor center, peritumoral white matter (PWM), and healthy white matter (healthy WM), in the contralateral hemisphere in the same location.

## 3. Results

**Figure 1:**
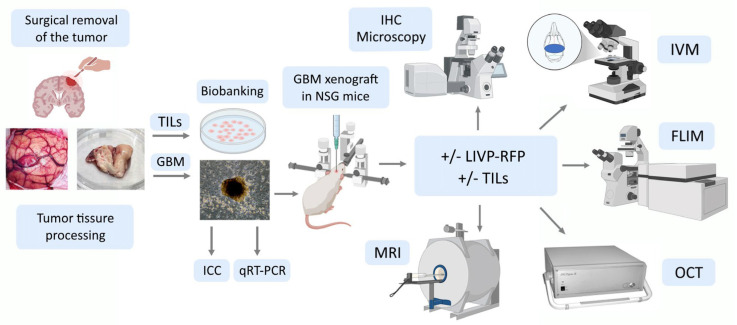
Experimental workflow: a stepwise assessment algorithm. TILs—tumor-infiltrating lymphocytes; GBM—glioblastoma multiforme; ICC—immunocytochemistry; IHC—immunohistochemistry; qRT-PCR—quantitative reverse transcription polymerase chain reaction; LIVP-RFP—vaccinia virus Lister strain from the Moscow Institute of Virus Preparation encoding the red fluorescent protein; IVM—in vivo microscopy; MRI—magnetic resonance imaging; OCT—optical coherence tomography; FLIM—fluorescence lifetime imaging microscopy.

Here, we developed a patient-derived xenograft culture-based platform that accurately replicates human tumor biology in immunodeficient mice, and might be used to predict therapeutic responses for individual patients. A stepwise assessment algorithm was designed: preparation of primary cultures from the surgically removed GBM tumor, qRT-PCR and ICC to assess heterogeneity of the primary cultures, engraftment of human glioblastoma cells in mice, magnetic resonance imaging (MRI) to study engrafted tumor, intravital microscopy to track targeted drug delivery, metabolic status assessment in glioma and periglioma zones following single exposure to OV or immunotherapy, and optical coherence tomography and histological and immunohistochemical analysis of mouse brain sections (Figure 1).

### 3.1. Primary Patient-Derived GBM Cells Express mRNA of Several Markers of Cancer Migration, Invasion, and Stemness in Different Media and Oxygenation Conditions

**Figure 2:**
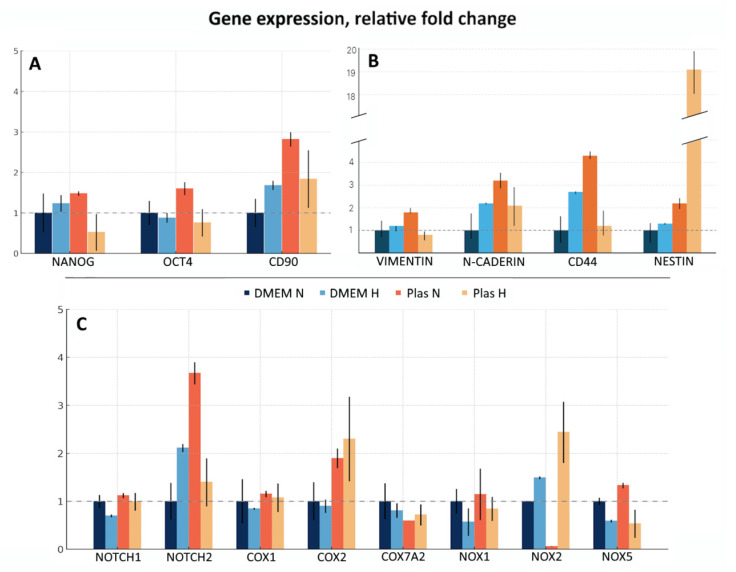
Gene expression of a panel of markers in GBM cells cultured in DMEM-F12 or Plasmax media under different oxygenation conditions. mRNA levels of a panel of the genes, including (**A**) genes encoding well-known markers of stem-like cells; (**B**) proteins involved in cancer cell migration, invasion, and interaction with extracellular matrix; (**C**) proteins involved in cancer cell signaling and metabo-lism were analyzed by qRT-PCR. Relative expression was calculated by the standard 2^−ΔΔCt^ method. Gene expression was normalized against GUSB and presented as fold change relative to DMEM N. Assays were run at least in duplicate. Data presented as the mean ± SE.

The composition of the culture medium as well as the oxygenation conditions influence cell phenotype and fate. In this study, we tested four culture conditions: in DMEM-F12 under “normoxia”, ~18.6% O_2_ (hereafter referred to as DMEM N); in DMEM-F12 under “hypoxia”, 5% O_2_ (hereafter referred to as DMEM H); in Plasmax under “normoxia” (hereafter referred to as Plas N); and in Plasmax under “hypoxia” (hereafter referred to as Plas H). The qRT-PCR was performed, assessing the expression of a broad panel of markers, namely genes encoding well-known markers of stem-like cells (Nanog, Oct4, CD90); proteins involved in cancer cell migration, invasion, and interaction with extracellular matrix (Vimentin, *N*-cadherin, CD44, Nestin; although Nestin is also a marker of stem-like cells); and proteins involved in cancer cell signaling and metabolism (Notch1, Notch2, Cox1, Cox2, Cox7A2, Nox1, Nox2, Nox5) (Figure 2).

For all subsequent experiments the Plas H culture condition was chosen as it is more physiologically relevant and based on the expression of all tested markers and remarkably high levels of Nestin mRNA. The ability of stem-like cells to initiate tumor formation upon transplantation into immunodeficient mice is their primary characteristic.

### 3.2. MRI Confirms Successful Engraftment of Human GBM PDX Cells in the Brain of NSG Mice

**Figure 3:**
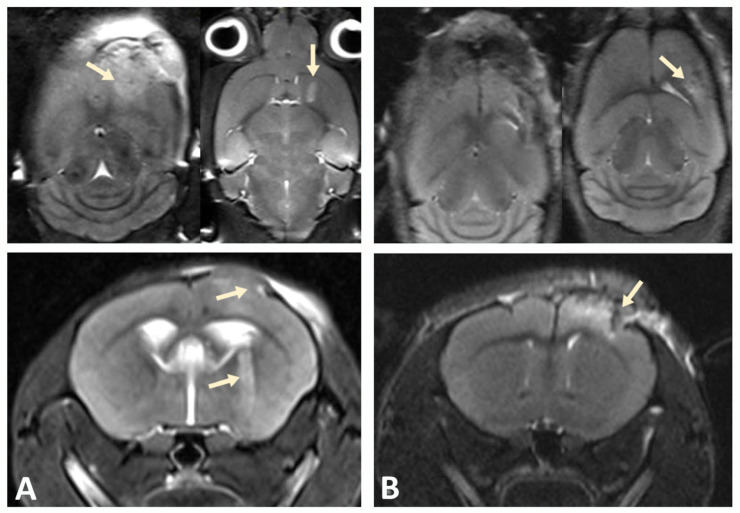
Tumor progression monitored by MRI. (**A**) On day 10, pronounced cortical growth of glioblastoma and a focus in the subcortical ganglia (indicated by arrows) were observed. (**B**) Implantation of GBM cells into the cortex of the hemispheres of NSG immunodeficient mice at the same time point was characterized by smaller tumor size.

A xenograft model was developed by stereotactic implantation of a suspension of glioblastoma (GBM) cells into the striatum of the right hemisphere of immunodeficient NSG mice. The MRI was used to assess tumor growth at the injection site and GBM cell migration after implantation in a mouse brain to validate successful establishment of the xenograft model. Scanning was performed in three projections to determine the direction of tumor cell migration within the mouse brain tissue. Tumor growth was observed in the injection zone, which confirmed the successful engraftment of human GBM xenograft cells and corresponded to the expected parameters of the model (Figure 3).

### 3.3. Immunochemical Analysis Demonstrates Astrogliosis Following Engraftment of PDX, Presence of Positive Markers for GBM Cells, and Absence or Low Number of Host Positive T, B, and NK Cells

**Figure 4:**
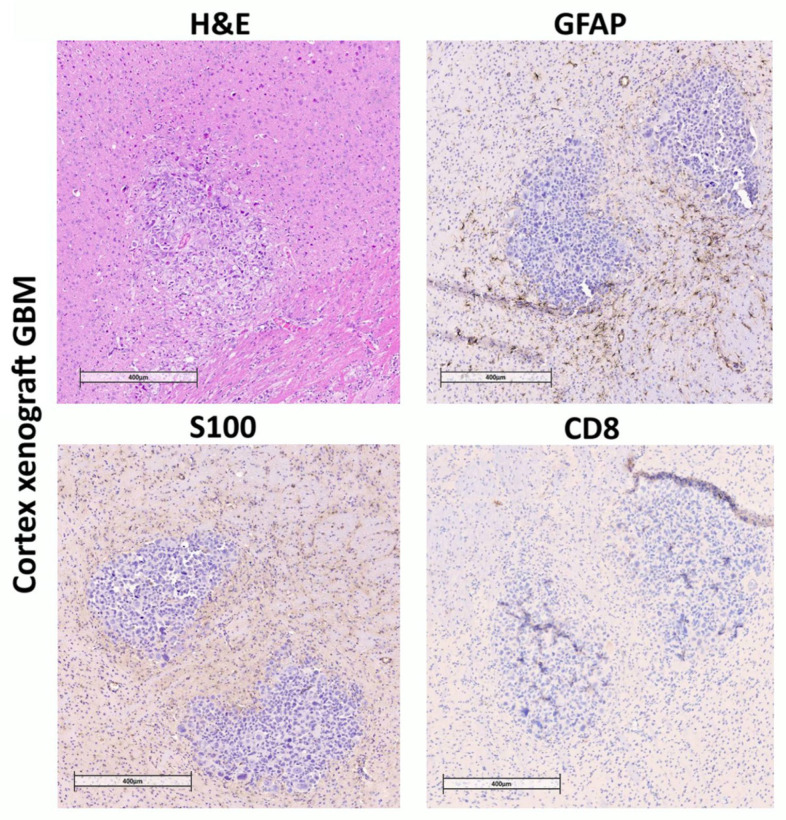
Immunohistochemical staining of brain sections from NSG mice inoculated with human GBM patient-derived xenografts into cortex. Mouse brain with human GBM xenograft tumor. Hematoxylin and eosin staining (H&E). Tissue infiltration by S-100-positive cells. Almost complete absence of CD8 lymphocytes, consistent with the immunological profile of NSG mice. Pronounced GFAP-positive glial infiltration around the tumor.

**Figure 5:**
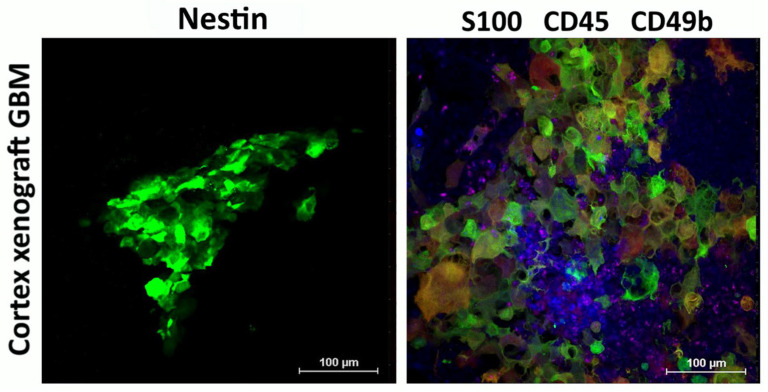
Immunophenotype of cortex xenograft GBM. Expression of Nestin (green) and S-100 (green) by human xenograft GBM cells engrafted in mouse brain, as well as recruitment of CD45+ (blue) and CD49b+ (purple) mouse cells to the tumor. Representative images.

After confirming tumor growth by MRI, a group of mice (see Materials and Methods) with implanted cortex or striatum PDX was used to assess the engraftment of human GBM cells in mouse brain sections. Immunohistochemical analysis of the obtained brain slices revealed reactive astrogliosis in the regions of stereotactic injection of human GBM cells, both in the cortex (Figure 4) and in the striatum of the right hemisphere (Figure 5). The astroglial reaction was determined by staining using anti-GFAP antibodies. Staining with antibodies recognizing S-100 (a protein commonly used as a diagnostic and prognostic marker of glioma) and CD8 (glycoprotein expressed by cytotoxic T lymphocytes) was also performed (Figure 4).

High levels of Nestin, S100, and Sox2, which were observed in primary GBM cultures, were retained in the engrafted human xenograft GBM (Figure 5).

**Figure 6:**
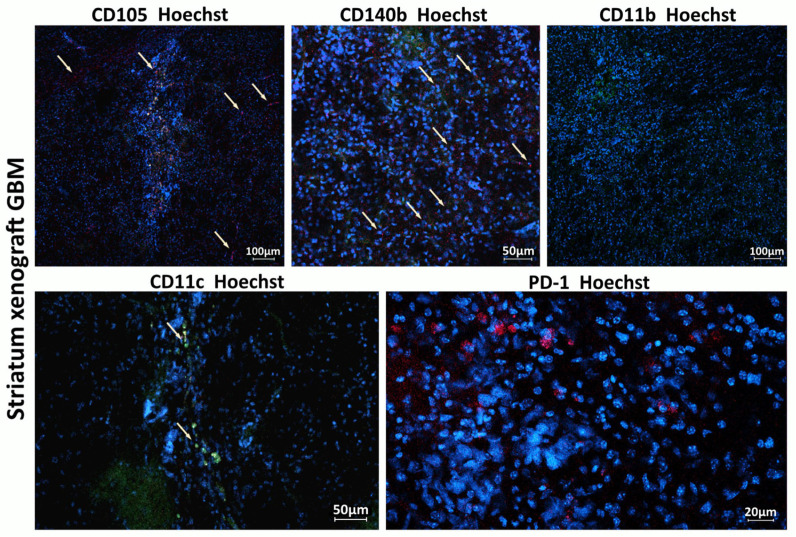
Host immune cells and cells involved in tumor angiogenesis in the TME of xenograft GBM. Staining for CD105+ (red), CD140b+ (red), CD11c (green), CD11b (green), and PD1 (red). Hoechst (blue) was used to stain nuclei. CD105 (endoglin) is a marker typically expressed by endothelial cells involved in tumor angiogenesis; CD140b (PDGFR-β) is a marker of pericytes and stromal cells supporting tumor vasculature; CD11c is a marker of dendritic cells involved in initiating immune response within the tumor; and CD11b is a marker of monocytes, macrophages, and microglia.

Recruitment of myeloid and lymphoid immune cells to the tumor, as well as presence of cells involved in tumor angiogenesis, was assessed using IVM and the intravenous injection of primary labeled antibodies against corresponding markers of these cells (Figure 6).

### 3.4. In Vivo Microscopy Confirms the Delivery of LIVP-RFP to the Human Xenograft GBM, and Characterizes Mouse Immune Milieu in the Tumor Microenvironment

**Figure 7:**
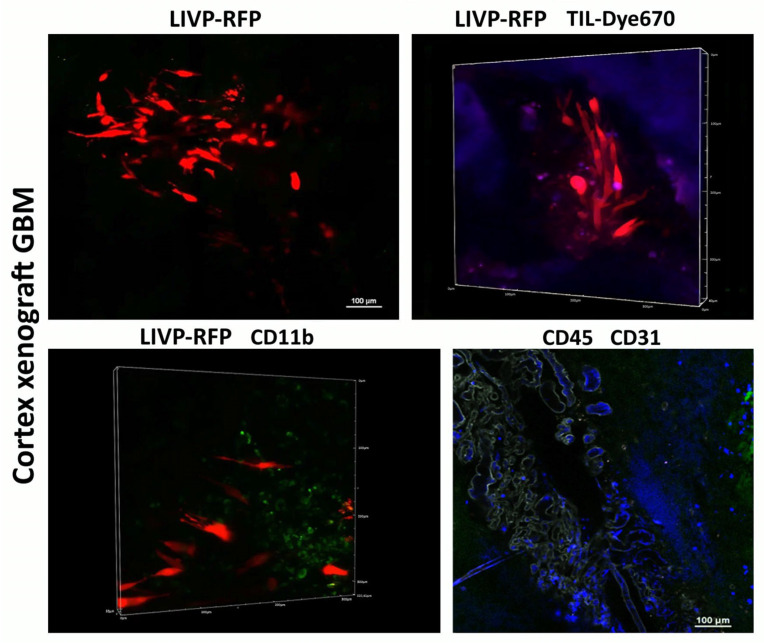
Immunophenotype of cortex xenograft GBM. Delivery of LIVP-RFP (red) and TILs (purple) to human xenograft GBM cells engrafted in mouse brain, as well as recruitment of CD45+ (blue), including CD11b+ (green) mouse cells across BBB CD31(grey) to the tumor. Representative images.

A xenograft in immunodeficient mice attracts myeloid cells to the microenvironment, but LIVP-RFP enhances the recruitment of CD45+ immune cells to the tumors, as confirmed by IVM. Delivery of viral particles to the tumor was visualized using RFP (Figure 7).

Following intravenous administration of the viral particle solution (LIVP-RFP) as specified in Materials and Methods, a signal from RFP is observed at 72 h in the cells of human glioblastoma implanted into the mouse brain cortex. Upon intravenous injection of TILs, the maximal signal from Dye670-labeled cells is detected in the peritumoral microenvironment at 48 h.

**Figure 8:**
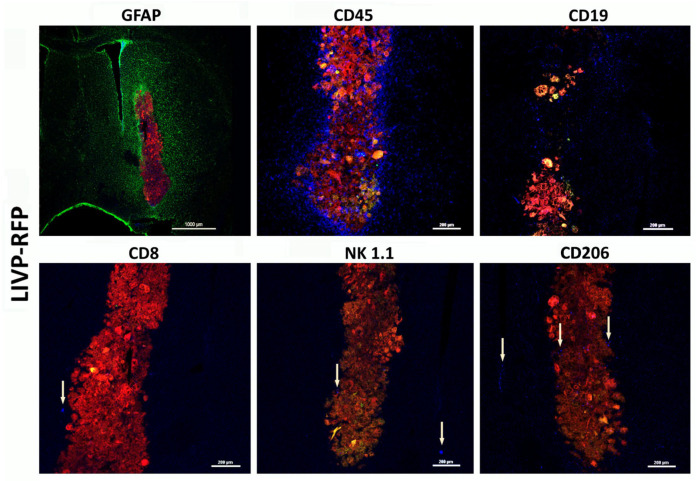
Astroglial reaction and immune cell infiltration following injection of LIVP-RFP in a xenograft GBM in NSG mouse model. Here, 48 h after stereotactic injection of LIVP-RFP (red) into the tumor, the mouse was perfused, and sections were stained with antibodies against GFAP (green), CD45+ (blue), CD19+ (blue), CD8+ (blue), NK1.1+ (blue), and CD206+ (blue).

**Figure 9:**
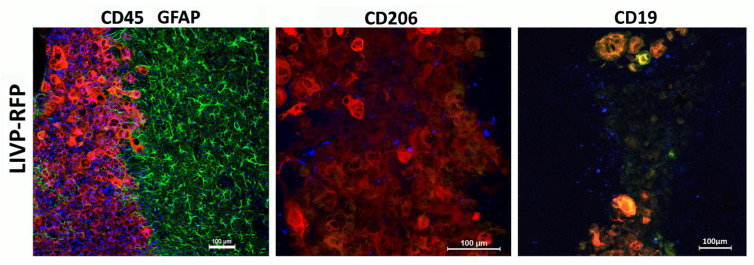
LIVP-RFP targets engrafted human xenograft GBM evoking recruitment of CD45+ mouse immune cells to tumors. LIVP-RFP (red) targets engrafted human xenograft GBM; CD45+ cells (blue), CD19+, and CD206+ (blue) tumor-associated macrophages infiltrate tumor and periglioma zone.

Despite active recruitment of CD45+ immune cells into the tumor microenvironment in response to LIVP-RFP therapy, only a few CD19+ cells were observed, with no more than two cells per field of view of NK1.1− and CD8+ cytotoxic lymphocyte-positive lymphocytes in the mouse (Figure 8 and Figure 9). This is consistent with the immunological profile of this mouse strain, which is known to have limited populations of these immune cell types. NK1.1 is a marker found on NK cells and some subsets of natural killer T cells (NKT) in mice, which play roles in innate immunity and anti-tumor responses. The low numbers of these cells are in line with the immunodeficient or immune-modified background of the mouse model used.

The recruitment of CD45+ cells to the virus-infected tumor and periglioma zone is observed. Astrocytes immediately become “reactive” in response to injury stimuli, which is a process called astrogliosis. Reactive astrocytes exhibit cellular hypertrophy and increased expression of GFAP, and can also act as atypical antigen-presenting cells. In this case, they release a diverse repertoire of signaling molecules, including several cytokines and chemokines, which modulate the immune response. Astroglial reaction and immune cell infiltration work together to manage the tumor, forming a complex inflammatory response that is both protective and potentially harmful over time. This dynamic interaction involves reciprocal signaling and physical changes to the brain environment. In our model, there was extensive astroglial scarring surrounding the tumor, whereas CD45+ cell infiltration decreased with increasing distance from the tumor core. We attribute this pattern to the characteristics of neovascularization and disrupted blood–brain barrier (BBB) within the tumor and peritumoral region, which change as the distance from the xenograft increases, accompanied by decreased permeability to high-molecular-weight agents and cells.

### 3.5. TILs Target Nestin and Sox2-Positive Stem-like Cells of Engrafted Human Xenograft GBM

**Figure 10:**
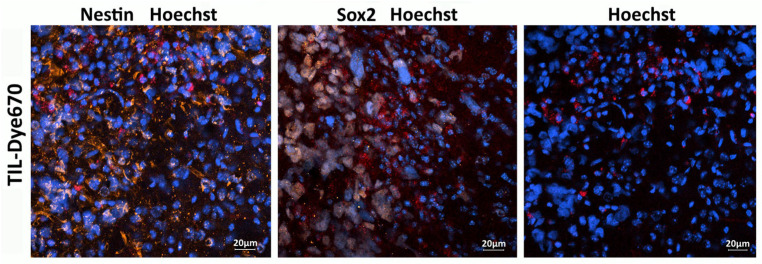
TILs (red) target Nestin (orange) and Sox2-positive stem-like cells (gray) of engrafted human xenograft GBM. Cell nuclei are stained blue (Hoechst 33258).

TILs, labeled with Dye 670, isolated from the patient’s biospecimen, were administered intravenously to mice bearing a GBM xenograft derived from the same patient. Confocal microscopy of brain sections performed 48 h post-injection detected human TILs within the tumor region and the periglioma zone (Figure 10).

### 3.6. FLIM of Xenograft in Brain Tissues of GBM NSG Mouse Model

In the present study, FLIM was performed on the brain tissue in the xenograft GBM NSG mouse model. All values of fluorescence decay parameters in the NAD(P)H channel are presented in Table 3.

**Figure 11:**
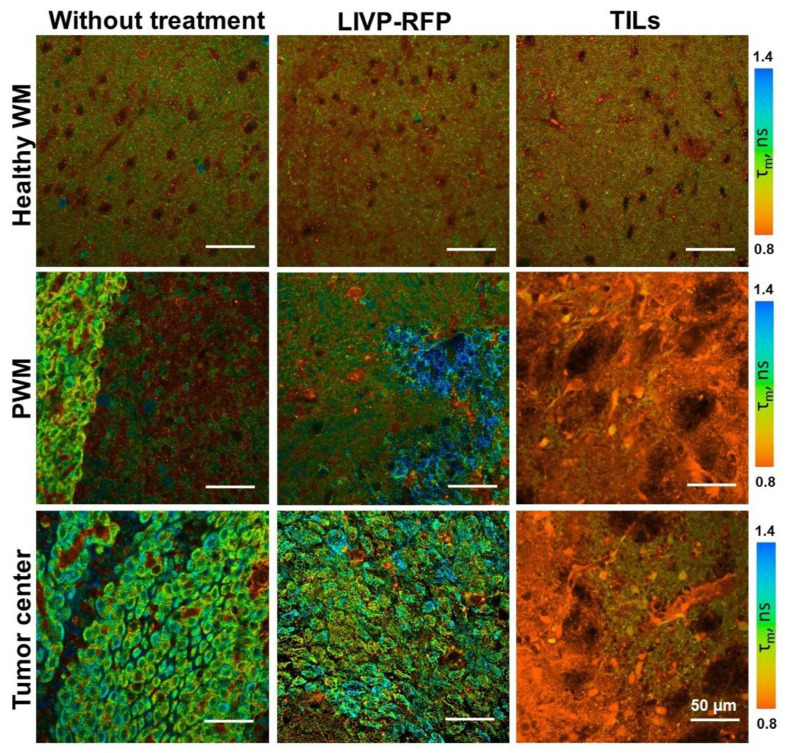
FLIM of brain tissues with xenograft GBM of control mice (Control), mice after oncolytic virus LIVP-RFP treatment, and mice after treatment with TILs. Representative images of brain tissue slices. Mean lifetime τm is shown (ex. 750 nm, em. 450–490 nm). Scale bar 50 µm.

**Figure 12:**
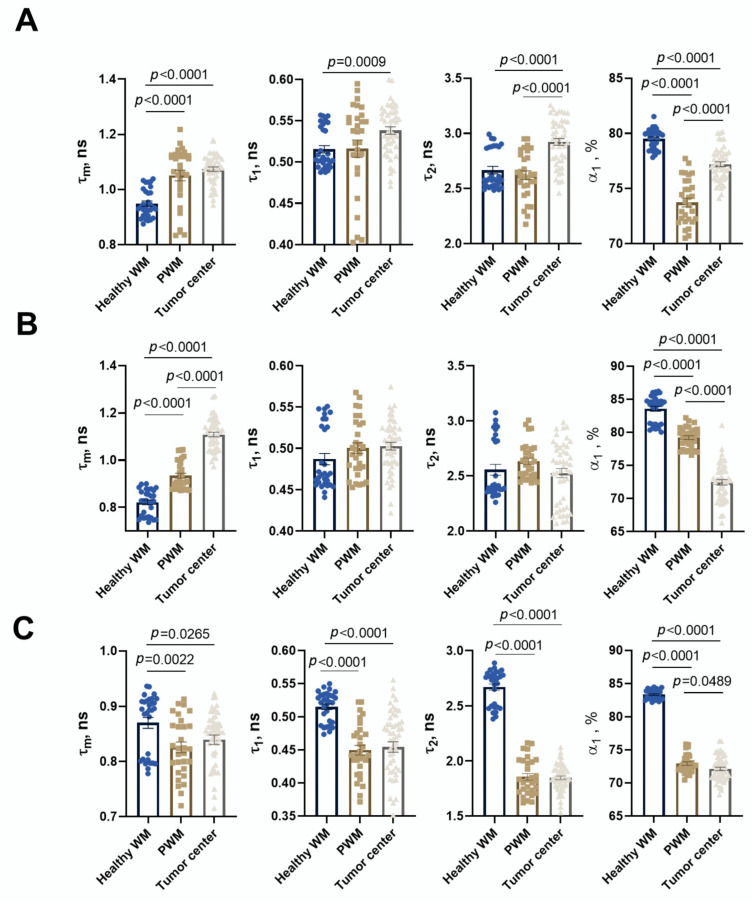
Quantification of the fluorescence decay parameters in brain tissues with xenograft GBM of control mice (**A**), mice after oncolytic virus LIVP-RFP treatment (**B**) and after TILs (**C**). Values of the mean fluorescence lifetime τm, the short fluorescence lifetime τ1, the long fluorescence lifetime τ2, and the relative contribution of short lifetime a1 are presented. Scatter dot plot displays the measurements for individual field of view/cells (dots) and the mean and SEM (*n* = 50–60 tumor cells or 10–12 ROI) (horizontal lines). Data distribution was assessed using the unpaired Student *t*-test. PWM—peritumoral white matter.

In healthy brain tissue without a tumor xenograft, the parameter values were τ1 0.52 ± 0.01 ns, τ2 2.67 ± 0.03 ns, α1 79.5 ± 0.2%, and τm 0.95 ± 0.01 ns. Tumor tissue demonstrated, on average, statistically significantly higher parameter values compared to a healthy brain: τ1 0.54 ± 0.01 ns (*p* = 0.0009), τ2 2.92 ± 0.03 ns (*p* < 0.0001), α1 77.2 ± 0.2% (*p* < 0.0001), and τm 1.07 ± 0.01 ns (*p* < 0.0001). Peritumoral white matter (PWM) differed from normal tissue in parameters a1 (α1 76.3 ± 0.4%, *p* < 0.0001) and τm (1.05 ± 0.02 ns, *p* < 0.0001) (Figure 11 and Figure 12A; Table 3). After oncolytic LIVP-RFP therapy, the difference between normal tissues and the tumor core was enhanced due to a decrease in the relative contribution of the short fluorescence lifetime component α1 (72.5 ± 0.4% vs. 83.6 ± 0.4% in normal tissue, *p* < 0.0001). This was reflected in the mean lifetime τm (1.11 ± 0.01 ns in tumor vs. 0.82 ± 0.01 ns in normal tissue, *p* < 0.0001). The tumor also differed from the PWM (α1 79.2 ± 0.3%, *p* < 0.0001; τm 0.93 ± 0.01 ns, *p* < 0.0001) (Figure 11 and Figure 12B; Table 1, Table S1: Fluorescence decay parameters of the mouse brain tissues with xenograft GBM in the NAD(P)H spectral channel.).

TIL-based therapy induced a pronounced decrease in parameter values compared to normal tissue for all fluorescence lifetime parameters: values in the tumor reached τ1 0.45 ± 0.01 ns, τ2 1.85 ± 0.02 ns, α1 72.2 ± 0.3% (*p* < 0.0001), and τm 0.95 ± 0.01 ns (*p* = 0.0265 vs. healthy brain). Peritumoral white matter had values close to those of the tumor and also differed significantly from normal tissues (τm *p* = 0.0022; τ1, τ2, α1 *p* < 0.0001) (Figure 11 and Figure 12C; Table 3).

**Figure 13:**
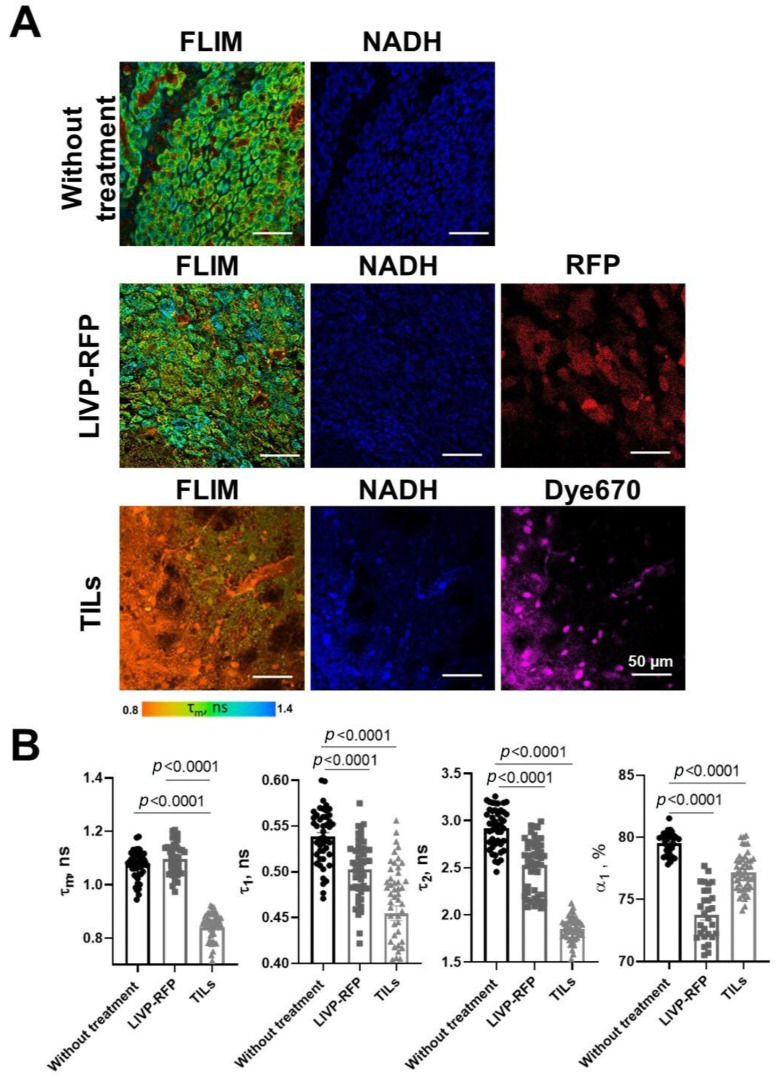
FLIM and confocal microscopy of xenograft GBM center in mice without treatment, after treatment with oncolytic virus LIVP-RFP, and after TIL treatment. (**A**) Representative FLIM images in NAD(P)H channel; mean lifetime τm is shown. Confocal images of NAD(P)H fluorescence (ex. 750 nm, em. 450–490 nm), RFP fluorescence (ex. 594 nm, em. 610 nm), and Dye670 (ex. 633, em. 670) fluorescence of brain tissue slices. Scale bar 50 µm. (**B**) Quantification of the fluorescence decay parameters in the xenograft GBM tumor center. Values of the mean fluorescence lifetime τm, the short fluorescence lifetime τ1, the long fluorescence lifetime τ2, and the relative contribution of short lifetime a1 are presented. Scatter dot plot displays the measurements for individual cells (dots) and the mean and SEM (*n =* 50–60 tumor cells or 10–12 ROI) (horizontal lines). Data distribution was assessed using the unpaired Student *t*-test.

Comparative analysis of tumors from untreated control mice versus from those treated with either oncolytic virus LIVP-RFP or TILs demonstrated significantly decreased fluorescence lifetime parameters in the virus-treated group, specifically in τ1, τ2, and α1 (*p* < 0.0001). The most pronounced reduction across all measured parameters (τm, τ1, τ2, α1; *p* < 0.0001) was observed in the TIL-treated group of mice (Figure 13B).

### 3.7. OCT of Xenografts in Brain Tissues of GBM NSG Mouse Model

**Figure 14:**
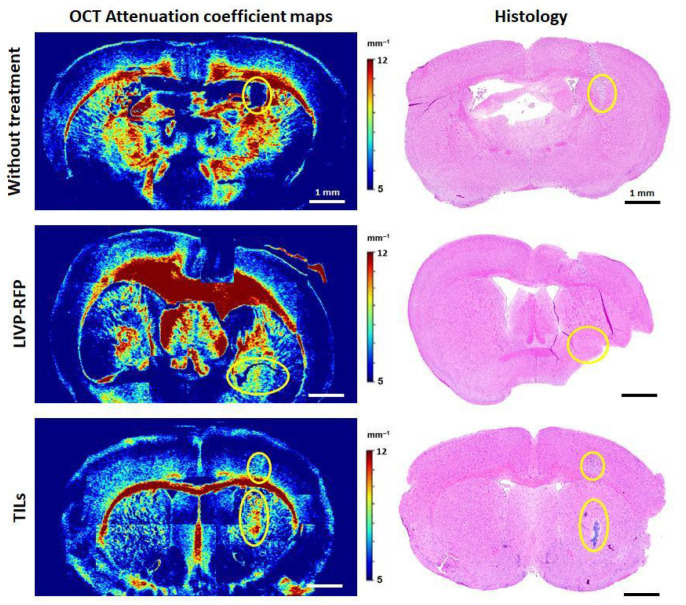
Attenuation coefficient maps and the corresponding histology of brain tissues with PDX GBM for mice without treatment, mice after LIVP-RFP therapy (LIVP-RFP), and mice after TIL therapy (TILs). Yellow ovals indicate the areas of tumor localization identified on OCT maps; the presence of tumor cells in these areas was confirmed by histology.

Color-coded maps of μ and the corresponding histology of brain tissues with xenograft GBM for mice without treatment, mice after LIVP-RFP therapy, and mice after TILs therapy are presented in Figure 14. In the group without treatment, in the color-coded maps of μ, localization of the growing tumor is clearly distinguishable from the surrounding brain structures; in this region, the attenuation coefficient μ is significantly reduced (3.78 ± 0.24 mm^−^^1^) compared to PWM (12.70 ± 0.40 mm^−^^1^, *p* < 0.0001) and healthy white matter (11.20 ± 0.60 mm^−^^1^, *p* < 0.0001). Moreover, the orderly pattern of myelin fibers and bundles (visible in red, yellow and bright green colors depending on the concentration and arrangement of myelin fibers) tends to be disrupted compared to the contralateral hemisphere. Morphologically, the xenograft GBM is presented by densely packed glioma cells. After therapy by LIVP-RFP, the xenograft GBM was reduced in size. In OCT attenuation coefficient maps, areas of treated tumor remained dark blue, which corresponds to low values of μ (2.77 ± 1.32 mm^−^^1^), even lower than untreated tumor (3.78 ± 0.24 mm^−^^1^). The PWM demonstrated decreased values of μ (8.01 ± 0.74 mm^−^^1^, *p* < 0.0001), which were statistically significantly lower than the values of healthy white matter in the lateral hemisphere (10.41 ± 0.35 mm^−^^1^).

An opposite optical effect was observed after TILs therapy: the tumor areas became brighter compared to the surrounding brain tissue. There was a sharp increase in the coefficient values (up to 9.17 ± 0.67 mm^−^^1^, *p* < 0.0001) compared to untreated tumors (3.78 ± 0.24 mm^−^^1^). Further, PWM had slightly reduced values of μ (6.88 ± 0.87 mm^−^^1^) compared to the same area in the contralateral hemisphere (7.10 ± 0.26 mm^−^^1^).

All values of the attenuation coefficient calculated for OCT images are presented in Table 3.

## 4. Discussion

The proper cell model for evaluating personalized therapy by autologous TILs, allogeneic therapy using modified or non-modified NK cells, and personalized selection of chemotherapeutic agents is the model recapitulating the heterogeneity of the patient’s tumor. The use of primary patient-derived GBM cultures, retaining cell heterogeneity of the patient’s tumor, allows more reliable and representative data to be obtained compared to those obtained using cell lines, which is particularly important for the development and testing of new personalized, patient-specific therapies. Human GBM cell lines, or patient-derived primary cultures—if grown in standard culture medium containing fetal animal or human serum—do not reflect the heterogeneity of human tumors and therefore do not fully resemble tumor behavior in xenograft models. Moreover, long-term culture results in clonal selection and genetic drift. An overview of 120 papers describing primary GBM cultures demonstrates that there are a variety of culture conditions used for primary GBM, and it is still unclear which media is ideal in such a context [25].

Replacement of “classical” media, in particular DMEM-F12 and RPMI, which do not replicate the metabolite composition present in blood plasma and cause non-physiological adaptations in cultured cells, with plasma-like media (HPLM and Plasmax) opens up new opportunities in GBM research [26,27]. Further, standard “normoxic” cell culture (~18.6% O_2_) is hyperoxic relative to physiological O_2_ levels in the brain or to hypoxic conditions inside the tumor. In our study we confirmed that culturing cells at a lower oxygen level better mimics in vivo conditions and helps maintain the stem cell markers and tumorigenic properties of primary cultures (Figure 2).

The *Il2rg* gene encodes the common gamma chain (γc), a receptor subunit essential for signaling of several cytokines in immune cells. Its loss in this strain results in significant elimination of NK cells, thus enhancing human cell engraftment in immunodeficient animal models of GBM. The lack/deficiency of the *Prkdc* gene, encoding the catalytic sub-unit of the DNA-dependent protein kinase (DNA-PKcs), impairs DNA double-strand break (DSB) repair and variable–diversity–joining rearrangement (V(D)J) recombination in developing lymphocytes. This, in turn, disrupts normal T and B lymphocyte development and leads to severe combined immunodeficiency (SCID) [28]. As a result, mice from the NOD *Prkdc* scid *Il2rg*em1/Smoc strain do not have fully functional T, B, and NK cells, which permits efficient engraftment of xenograft tumors and provides a good platform for evaluating cell-based immunotherapy of human GBM xenografts.

Here, we established an orthotopic xenograft GBM NSG mouse model. The xenografts exhibited high infiltrative activity, migrating along the white matter tracts and spreading through the corpus callosum. This is consistent with previously reported well-defined pathways of xenotransplanted GBM migration in the mouse brain [29].

The characteristics of the original patient’s tumor are preserved during xenotransplantation into immunodeficient mice, allowing the study of personalized and allogeneic therapy in this model using TILs, autologous CTL, or genetically modified CAR-T or CAR [30].

The brain has always been considered an immune-privileged organ due to the existence of the BBB—a barrier created by micro-vascular endothelial cells connected by tight junctions and adherens junctions, basement membrane, and glial cells which surround capillaries. However, patients with GBM are characterized by BBB’s vascular fragility and “leakage” [31], making it somewhat permeable for immune cells.

Further, the brain has its own immune system, including microglia (resident brain immune cells), macrophages, innate immune cells, and small populations of T and B lymphocytes. The role of the brain immune system in oncogenesis has been shown to be ambiguous, particularly the reversible pro-tumor and immunosuppressive effects of microglia and macrophages [32]. These cells are able to cross the BBB in GBM patients [31]. Additionally, a phenomenon has been observed where activation of resident microglia in response to modeling can cause rejection of low-grade gliomas in the brains of nude mice [33,34].

In this work, we demonstrated that a heterogeneous human xenograft GBM culture is capable of engrafting in the brain of NSG mice while simultaneously stimulating active recruitment of resident microglia and macrophages into the tumor both before and after viral therapy. This indicates a “cross-talk” between the xenograft GBM and host immune system in our model, perhaps evoking certain changes both in the xenograft and in the host, leading to a “mouse model-specific” evolution of the xenograft. In particular, using IVM, we demonstrated active infiltration of xenograft GBM by macrophages.

Programmed death-1 (PD-1) is an immune checkpoint receptor upregulated on activated T cells and some other immune cells within the tumor microenvironment, playing a key role in immune tolerance towards a tumor. Previous works report expression of PD-1 by tumor-associated macrophages (TAMs), both in humans and in mice [35], and indicate that they have a type 2 phenotype [36]. The studies by Kono et al. and by Jiang et al. demonstrated the relationship between the presence of PD-1-positive TAM and clinicopathological indicators such as poor prognosis/poor clinical outcome in cancer [36,37]. It is believed that PD-1 expression by TAM negatively correlates with their phagocytic activity toward tumor cells, whereas in vivo blockade of PD-1/PD-L1 enhances macrophage phagocytosis, slows tumor growth, and prolongs survival in mouse models in a macrophage-dependent manner [35]. In our study, we also detected PD-1 expression by immune cells recruited to the tumor. Thus, this data suggest that our xenograft model might be suitable for future studies of PD-1/PD-L1 therapy and factors modulating its efficacy [38]. We have also demonstrated that in our model LIVP-RFP is capable of crossing the BBB (in case of intravenous injection) and targeting GBM xenografts (in the case of both intratumoral and intravenous routes of delivery), as assessed by red fluorescence. Further, we have shown that TILs can also reach the xenograft via both routes of delivery.

It is known that integrins CD49a, CD49b, CD49d, and CD49e are expressed at low levels by macrophage-activated cells and dendritic cells, with the highest expression observed on monocytes. The majority of the remaining immune cells in the NSG mice are neutrophils and monocytes, detectable in peripheral blood (as per the Jackson Laboratory’s NSG mice portfolio). CD49b can also be a marker of different hematopoietic stem/progenitor cell (HSPC) populations. We observed a small number of CD49b-positive cells recruited to the GBM xenograft site, and hypothesize that viral infection enhanced the recruitment of these cells.

Detection methods such as optical metabolic imaging and optical coherent tomography, which do not require specific dyes for detection, are of greater interest as a screening option aimed at clinical application. Previously, we conducted a study where these methods were applied to a glioblastoma xenograft model in Nude/Balb mice. However, this model is not suitable for studying the effectiveness of autologous lymphocytes, allogeneic cells, or modified killer cells, and secondly, the work was carried out using a human glioblastoma cell line grown under standard conditions (normoxia, DMEM-F12, FBS).

Our FLIM results demonstrate the statistically significant increase in τ1, τ2, and τm in tumor tissue compared to healthy brain tissues, which is consistent with previous studies provided on glioma patients’ brain samples [39]. In this study, the authors hypothesize that the long fluorescence lifetime τ2, which corresponds to protein-bound NAD(P)H, increases or decreases based on the activity of enzymes that bind to it. These enzymes are crucial for cell metabolism as they direct carbon through metabolic pathways to the mitochondrial respiratory chain [40]. Notably, the significant differences found in the peritumoral white matter (PWM) for α1 and τm suggest that FLIM can detect field effects or early infiltrative changes, a finding supported by research on tumor microenvironment remodeling. The α1 parameter, relative contribution of the short component of fluorescence lifetime, typically correlates with glycolytic activity [41]. Furthermore, the therapeutic efficacy of both oncolytic vaccinia virus LIVP-RFP and TILs was quantitatively reflected in a pronounced reduction in fluorescence lifetime parameters within the tumor center. The fact that TIL-based therapy induced the most substantial decrease across all parameters (τm, τ1, τ2, α1) underscores the potential of FLIM not only for diagnostics but also for non-invasive monitoring of treatment response.

OCT has similar potential for non-invasive assessment of GBM response to various therapies as well as the detection of the tumor among the surrounding white matter of the brain. Thus, it is not surprising that the interest in using OCT to study brain gliomas in animal models remains high. For example, in a recent paper by Nguyen-Hoang et al. [42], OCT and OCT angiography were applied to study the growth of brain tumors in C57BL/6 mice. Unlike our deep tumor grafting into the brain (so that the growing tumor is surrounded by myelin fibers), the authors introduced glioma GL261 cells superficially into the cerebral cortex and used homogeneity and variance of the OCT backscattered intensity for quantitative analysis of OCT images. Katta N. with coauthors [43] used OCT image-guided laser surgical system for excision of brain tumors in vivo in a murine xenograft model. Human glioma cells (U251-luc-RFP) were used to create a tumor node at a depth of 0.3–1.15 mm, allowing visualization of the entire tumor using OCT. In structural OCT images, tumor areas were identified based on a threshold attenuation coefficient of less than 5.7 mm^−^^1^. This is consistent with our findings of a low attenuation coefficient in a growing murine xenograft brain cancer model. After the tumor node and surrounding vasculature were imaged by the OCT imaging system, the tumor underwent surgical removal utilizing Tm wavelength for coagulation and Er:YAG for ablation, also with control of the resection volume using OCT. Therefore, the OCT technology is very promising for visualizing the margins of glioma tumor growth, assessing the completeness of tumor resection, and monitoring the tumor response to different therapeutic agents.

In the current study, for the first time we measured the attenuation coefficient (μ) of an orthotopic xenograft GBM. The low values of μ = 3.78 ± 0.24 mm^−^^1^ were obtained, which is consistent with other glioma models. For example, for rat glioblastoma 101.8 without necrosis, μ was equal to 3.72 [3.51; 4.17] mm^−^^1^ [44], and in the case of patient glioma samples, for astrocytoma Grade I–III, μ = 3.0 [2.6; 3.5] mm^−^^1^, and for glioblastoma Grade IV without necrotic areas, μ = 3.15 [2.6; 4.2] mm^−^^1^ [45]. Further, we showed that the μ values of healthy white matter were 11.20 ± 0.60 mm^−^^1^, while in the immediate vicinity of the tumor the values were slightly higher (12.70 ± 0.40 mm^−^^1^), which may indicate compression of the myelin fibers by the tumor node. A similar phenomenon was described by Achkasova K et al. [23], where cases of displacement and compression of white matter due to tumor growth in rat glioma models were described.

In addition to calculating the OCT attenuation coefficient values for the xenograft GBM during its natural growth, we constructed color-coded OCT attenuation coefficient maps and obtained values after oncolytic virotherapy by LIVP-RFP and TIL therapy. Differences in optical properties between these two treatments were obvious: in the case of LIVP-RFP treatment, the tumor retained a low attenuation coefficient (2.77 ± 1.32 mm^−^^1^), while TIL treatment resulted in a sharp increase in μ values up to 9.17 ± 0.67 mm^−^^1^, 2.4 times higher than pre-therapy values. Such contrasting changes may be due to different mechanisms of action of these therapeutic agents. The aforementioned effect observed in the case of LIVP-RFP can be explained by enhanced infiltration of the tumor by immune cells. The sharp increase in the coefficient values (up to 9.17 ± 0.67 mm^−^^1^, *p* < 0.0001) compared to untreated tumor (3.78 ± 0.24 mm^−^^1^) in the case of TIL therapy may be due to cell death of most tumor cells.

In this series of experiments utilizing OCT and calculating μ, we, for the first time, demonstrated the multidirectional optical changes in tumor tissues in response to LIVP-RFP and TIL therapy, as well as a decrease in μ values in PWM. The results show that OCT can be used for distinguishing xenograft GBM localization among different brain structures and for evaluating various morphological states of tumor cells as well as peritumoral white matter after therapy, which indicates the potential of the OCT application for non-invasive monitoring of the GBM xenograft response to the aforementioned therapies in the NSG mouse model.

Of note, using FLIM and OCT enables the study of GBM xenografts without the need for fluorescent protein labeling of the tumor cells, because these techniques rely on intrinsic optical properties of tissues. This is of particular importance, because introduction of the fluorescent proteins to the tumor xenograft can affect its immunogenicity and other properties [46,47]. Thus, using “label-free” xenografts in combination with FLIM and OCT enhances the translational relevance of preclinical GBM models.

## 5. Conclusions

The appropriate choice of animal model should depend on the specific research question for the optimal transition from laboratory studies to clinical practice. The xenograft GBM animal model developed in this study using the immunodeficient NSG mice proved to be a suitable tool for evaluating TIL immunotherapy and therapy by OV. We demonstrated that heterogeneous short-term primary cultures of patient-derived GBM can engraft in these mice and evoke active recruitment of resident microglia and macrophages to the tumor site. It was determined that the VV strain LIVP-RFP specifically targets xenograft GBM in this model. Metabolic changes in GBM in response to the therapy by application of OV (in particular, of LIVP-RFP) and TILs can be evaluated in this model by FLIM.

## Figures and Tables

**Table 1:** The scheme of random assignment of mice to the cortex and striatum groups in the experiment.

Stereotaxic Coordinates	Intracranial Implantation hGBM Cells	MRI	ICH and Histology Before Treatment	OV or TILs	IVM IV	FLIM ST	OKT ST	ICH After Treatment	Histology
Cortex AP − 2.0, D 1.5, V 1.2.	11	11	2	OV	4	-	-	-	4
				TILs	4	-	-	-	4
				Control	1	-	-	-	1
Striatum AP + 1, D 1.5, V 2.5.	12	12	3	OV	-	3	3	3	3
				TILs	-	3	3	3	3
				Control	-	3	3	3	3

**Table 2:** The scheme of the experiment after treatment in vivo.

Group	Administration Way	Virus Dose	TILs Dose	Administration Scheme	Study
**Cortex** AP − 2.0, D 1.5, V 1.2.	Intratumoral (i.t.)	1 × 10^6^ PFU	1 × 10^6^ cells	1 injection	IVM
	Intravenous (i.v.)	1 × 10^9^ PFU	2 × 10^6^ cells	1 injection	IVM
**Striatum** AP + 1, D 1.5, V 2.5.	Intratumoral (i.t.)	1 × 10^6^ PFU	1 × 10^6^ cells	1 injection	FLIM OCT
**Control** Cortex					IVM
**Control** Striatum					FLIM OCT

**Table 3:** Values of attenuation coefficient μ of the mouse brain tissues with PDX GBM. Mean ± SEM (*n* = 4–11 regions of interest).

μ	Without Treatment	LIVP-RFP Therapy	TIL Therapy
μ (Healthy WM), mm^−1^	11.20 ± 0.60	10.41 ± 0.35	7.10 ± 0.26
μ (PWM), mm^−1^	12.70 ± 0.40	8.01 ± 0.74	6.88 ± 0.87
μ (Tumor center), mm	3.78 ± 0.24 *^,^**	2.77 ± 1.32 *^,^**	9.17 ± 0.67 **

* *p* < 0.05 from healthy WM, ** *p* < 0.05 between PWM and tumor zone (*p*-value was assessed with unpaired Student’s *t*-test).

## Data Availability

The data presented in this study are available upon reasonable request from the corresponding author.

## Supplementary Materials

The following supporting information can be downloaded at https://www.mdpi.com/article/10.3390/biomedicines13122977/s1: Table S1: Fluorescence decay parameters of the mouse brain tissues with xenograft GBM in the NAD(P)H spectral channel.

## Abbreviations

BBB	Blood–brain barrier
CAR	Chimeric antigen receptor
CD	Cluster of differentiation
cDNA	Complementary deoxyribonucleic acid
CI	Cancer immunotherapy
DMEM	Dulbecco’s modified eagle medium
DNA	Deoxyribonucleic acid
DPBS	Dulbecco’s phosphate-buffered saline
FBS	Fetal bovine serum
FLIM	Fluorescence-lifetime imaging microscopy
GBM	Glioblastoma multiforme
GFAP	Glial fibrillary acidic protein
HPML	Human plasma-like medium
ICC	Immunocytochemistry
IHC	Immunohistochemistry
IL	Interleukin
IVM	Intravital microscopy
LIVP	Lister strain from the Moscow Institute of Virus Preparation
MRI	Magnetic resonance imaging
mRNA	Messenger ribonucleic acid
NAD(P)H	Reduced nicotinamide adenine dinucleotide phosphate
NADP	Nicotinamide adenine dinucleotide phosphate
NK	Natural killer
NKT	Natural killer T cells
NOD	Non-obese diabetic
NSG	NOD-*Prkdc* scid *Il2rg*em1/Smoc
OCT	Optical coherence tomography
OV	Oncolytic virus
PBS	Phosphate-buffered saline
PCR	Polymerase chain reaction
PD-1	Programmed cell death 1
PD-L1	Programmed cell death ligand 1
PDGFR-β	Platelet-derived growth factor receptor β
PDX	Patient-derived xenograft
PECAM-1	Platelet endothelial cell adhesion molecule 1
PFU	Plaque-forming units
PTEN	Phosphatase and tensin homolog
PWM	Peritumoral white matter
qRT-PCR	Quantitative reverse transcription polymerase chain reaction
RFP	Red fluorescent protein
RNA	Ribonucleic acid
SCID	Severe combined immunodeficiency
TAM	Tumor-associated macrophage
TILs	Tumor-infiltrating lymphocytes
TME	Tumor microenvironment
VV	Vaccinia virus
