# Supplementary material for: Multiparametric Detection of Effects of TILs and Oncolytic Virotherapy on Xenograft Mouse Model of Glioblastoma
Source: Biomedicines. 2025 Dec 4;13(12):2977. doi: 10.3390/biomedicines13122977 (PMC12730700; doi:10.3390/biomedicines13122977)
Supplement: Supplementary file 1 [file biomedicines-13-02977-s001.zip › biomedicines-3963926-supplementary.pdf]

Supplementary Table 1

**Brain tissue types**	**τ_m, ns**	**τ_1, ns**	**τ_2, ns**	**a_1, %**	**a_2, %**
**Control mice**					
**Healthy brain**	0,95±0.01	0,52±0.01	2,67±0.03	79,5±0.2	20.4±0.2
**PWM**	1.05±0.02*	0.52±0.01	2.62±0.04	73.7±0.4*	26.3±0.4*
**Tumor center**	1.07±0.01*	0.54±0.01*	2.92±0.03*, **	77.2±0.2*, **	22.8±0.2*, **
**Mice after oncolytic Vaccinia Virus therapy**					
**Healthy brain**	0,82±0.01	0,49±0.01	2,56±0.05	83,6±0.4	16.4±0.2
**PWM**	0,93±0.01*	0.50±0.01	2.63±0.03	79.2±0.3*	20.8±0.3*
**Tumor center**	1.11±0.01*, **	0.50±0.01*	2.53±0.04*	72.5±0.4*, **	27.5±0.4*, **
**Mice after TILs therapy**					
**Healthy brain**	0,87±0.01	0,52±0.01	2,67±0.03	83,3±0.1	16.7±0.1
**PWM**	0,83±0.01*	0.45±0.01*	1,86±0.03*	73±0.3*	27±0.3*
**Tumor center**	0,84±0.01*	0.45±0.01*	1,85±0.02*	72.2±0.3*, **	27.8±0.3*, **
